# The Mucosae-Associated Epithelial Chemokine (MEC/CCL28) Modulates Immunity in HIV Infection

**DOI:** 10.1371/journal.pone.0000969

**Published:** 2007-10-03

**Authors:** Eleonora Castelletti, Sergio Lo Caputo, Louise Kuhn, Manuela Borelli, Johanna Gajardo, Moses Sinkala, Daria Trabattoni, Chipepo Kankasa, Eleonora Lauri, Alberto Clivio, Luca Piacentini, Dorothy H. Bray, Grace M. Aldrovandi, Donald M. Thea, Francisco Veas, Manuela Nebuloni, Francesco Mazzotta, Mario Clerici

**Affiliations:** 1 Department of Preclinical Sciences, Laboratorio Interdisciplinare Technologie Avanzate (LITA) Vialba, University of Milano, Milano, Italy; 2 Infectious Diseases Clinic, S.S. Annunziata Hospital, Antella, Firenze, Italy; 3 Gertrude H. Sergievsky Center, Department of Epidemiology, Mailman School of Public Health, Columbia University, New York, New York, United States of America; 4 Laboratory of Retroviral and Molecular Immunology, Research Institute for Development, IRD/UR178, Montpellier, France; 5 Lusaka District Health Management Team, Lusaka, Zambia; 6 University Teaching Hospital, University of Zambia, Lusaka, Zambia; 7 Pathology Unit, Department of Clinical Sciences, University of Milano, Milano, Italy; 8 ImmunoClin, London, United Kingdom; 9 Department of Pediatrics, University of Southern California, Los Angeles, California, United States of America; 10 Center for International Health and Development, Boston University School of Public Health, Boston, Massachusetts, United States of America; 11 Department of Biomedical Sciences and Technologies, University of Milano, Milano, Italy; University of California at San Francisco, United States of America

## Abstract

**Background:**

CCL28 (MEC) binds to CCR3 and CCR10 and recruits IgA-secreting plasma cells (IgA-ASC) in the mucosal lamina propria (MLP). Mucosal HIV-specific IgA are detected in HIV-infection and exposure. The CCL28 circuit was analyzed in HIV-infected and-exposed individuals and in HIV-unexposed controls; the effect of CCL28 administration on gastrointestinal MLP IgA-ASC was verified in a mouse model.

**Methodology/Findings:**

CCL28 was augmented in breast milk (BM) plasma and saliva of HIV-infected and –exposed individuals; CCR3+ and CCR10+ B lymphocytes were increased in these same individuals. Additionally: 1) CCL28 concentration in BM was associated with longer survival in HIV vertically-infected children; and 2) gastro-intestinal mucosal IgA-ASC were significantly increased in VSV-immunized mice receiving CCL28.

**Conclusions:**

CCL28 mediates mucosal immunity in HIV exposure and infection. CCL28-including constructs should be considered in mucosal vaccines to prevent HIV infection of the gastro-intestinal MLP via modulation of IgA-ASC.

## Introduction

Migration of IgA-secreting plasma cells into the mucosal lamina propria (MLP) was recently shown to be correlated with specific chemokines and chemokine receptors [1]–[13]. Thus, IgA-expressing plasma blasts and plasma cells (IgA-ASC) are characterized by a number of surface receptor proteins, including CCR9, CCR3, and CCR10, that are ligated by specific chemokines. In particular, it has been shown that CCR9 ligates the CC chemokine CCL25 (also known as thymus-expressed chemokine, or TECK) [4], [10], whereas both CCR10 and CCR3 bind a second CC chemokine named CCL28 (also known as mucosae-associated epithelial chemokine, or MEC) [1], [5], [10], [12]. These interactions induce the migration and the recruitment of IgA-ASC in the MLP. CCL25 was shown in a mouse model to mostly attract a subpopulation of IgA-ASC associated with the small intestine [4], [6], [10]. CCL28 is more widely expressed and potently chemoattracts IgA-ASC originating from diverse mucosal lymphoid organs, as well as from intestinal and extra intestinal tissues, in every studied mucosal effector site-including the mammary and salivary glands-both in mice and humans [1]–[7], [9]–[12], [14], [15]. Thus, the CCL28-CCR10/CCR3 circuit is considered to be a unifying system that plays a major role in the homing of plasma blasts and plasma cells at mucosal effector sites [3], [5], [13]. Interestingly, these chemotactic abilities are limited to IgA-ASC as the migration of neither IgM nor IgG ASC is stimulated by the CCL28-CCR10/CCR3 or the CCL25-CCR9 systems [1]–[13]. The CCL28-CCR10/CCR3 circuit is also endowed with other interesting peculiarities. Thus, CCL28 has a potent antimicrobial activity directed toward both Gram-positive and Gram-negative microorganisms [3]. Additionally, this chemokine is expressed by bone marrow stromal cells, suggesting that the interaction of CCL28 with CCR10+/CCR3+ B cells may contribute to the integration between the mucosal and the systemic immune responses [3].

High concentration of mucosal HIV-specific IgA have repeatedly been observed in HIV-infected individuals [16]–[19] and have also been suggested to characterize HIV sexually-exposed but uninfected individuals (Exposed Seronegatives or ESN) [20]–[23]. Thus, HIV-specific IgA were described to be present in saliva, cervico-vaginal secretions, and breast milk from both HIV-infected and ESN individuals in Europe, Asia and Africa by most [20]–[24], but not all [25] authors. To investigate whether the presence of high concentrations of mucosal HIV-specific IgA could be explained by an upregulation of the chemokine circuits involved in mucosal homing of IgA-ASC, we first measured CCL25 and CCL28 in breast milk of a well-characterized cohort of HIV-infected breast feeding mothers from Zambia. Results showing a significantly augmented concentration of CCL28 in HIV IgA-positive milk encouraged us to analyze CCR3-, CCR9-, and CCR10-expressing cells as well as the mucosal concentration of CCL25 and CCL28 in ESN subjects and their HIV-infected partners. Results were compared to those seen in HIV-unexposed individuals. Finally, as it was recently shown that the privileged site of HIV replication during primary infection is the gastro-intestinal (GI) mucosa, and IgA could protect this mucosa, we verified whether the administration of CCL28 would result in a significant modulation of IgA in the GI tract using an animal model undergoing immunization with a VSV construct in the presence/absence of CCL28. Results herewith indicate a pivotal role for CCL28 in the modulation of mucosal immunity in HIV exposure and infection and suggest a usefulness of CCL28-containing adjuvants in the development of HIV vaccines.

## Materials and Methods

### Study population

Breast milk samples were collected from 65 HIV-infected women enrolled in a breastfeeding study in Lusaka, Zambia [26]. The samples were selected to include 15 women who transmitted and 50 women who did not transmit HIV to their infant. Milk samples were collected by manual expression between 1 and 5 weeks post-partum. Milk samples from 9 uninfected Zambian women were included as controls. All women signed informed consent to participate.

Blood samples and saliva were collected from 39 HIV-exposed but uninfected heterosexual partners of HIV-infected individuals (ESN)(27 females; 12 males). The inclusion criteria for the ESN was a history of multiple unprotected sexual episodes (with the same HIV-seropositive partner) for ≥4 years at the time of enrollment, with at least 8 episodes of at-risk intercourse within the 4 months before study entry, and an average of 30 reported unprotected sexual contacts per year (range 18 to >100). Forty age-matched HIV-infected patients (HIV+) and 30 age- and sex-matched healthy volunteer controls (HC), without any known risk factor for HIV-infection, were studied as well. All ESN and HIV-infected patients underwent in-depth clinical and laboratory evaluation that did not reveal any concomitant infectious or gynecologic problems. All individuals (ESN, HIV+, and HC) had been longitudinally followed for >4 years before the study by the Department of Obstetrics and Gynecology, Santa Maria Annunziata Hospital, Florence. This allowed us to exclude from the study ESN and HC in whom sexually transmitted diseases or any other pathology was reported during that time period. Each subject signed an informed consent form that was approved by the Ethical committee of the S. M. Annunziata Hospital.

### Breast milk preparation

All milk was processed within 4 hours of collection. Milk was centrifuged at 400g and the cell pellet removed. The supernatant and lipid portions were mixed together before aliquoting and were stored at −70°C until use.

### Blood sample collection and separation of PBMC

Whole blood was collected by venipuncture in Vacutainer tubes containing EDTA (ethylenediaminetetraacetic acid) (Becton Dickinson, Rutherford, NJ). Peripheral blood mononuclear cells (PBMC) were separated on lymphocyte separation medium (Organon Teknica, Durham, NC) and washed twice in phosphate-buffered saline (PBS; Organon Teknica). The number of viable leukocytes was determined by trypan blue exclusion. All analyses were performed on freshly collected cells.

### Immunophenotypic analysis

Surface receptors were evaluated using 50 µl of EDTA peripheral blood. Cells were incubated for 30 min at 4°C with the following flourochrome-labeled mAbs: CD19- R-phycoerythrin-Cyanine 5 Tandem–PE-Cy5 (mouse IgG1 isotype, Beckman Coulter, Fullerton CA), CCR3-Phycoerythrin and CCR9-Fluorescein (mouse IgG2a isotype, R&D Systems, Minneapolis, MN). After incubation, erythrocyte lysis and fixation of marked cells was performed using the Immuno-Prep EPICS Kit (Coulter Electronics) and Q-prep Work Station (Coulter Electronics, Miami Lakes, FL).. CCR10 expression was evaluated by indirect immunofluorescence staining on PBMC: by incubating 250,000 cells with a goat anti human CCR10 (diluted 1∶25) (Capralogics Inc., Hardwick MA). After 30 min at 4°C, the cells were washed and further incubated for 30 min at 4°C with 10 µl porcine anti-goat IgG conjugated with PE (R&D Systems, Minneapolis, MN). After this incubation cells were washed and fixed in 1% formaldehyde. Cytometric analyses were performed using an EPICS XL flow cytometer (Beckman-Coulter Inc., Miami, FL) equipped with a single 15 mW argon ion laser operating at 488 nm interfaced with 486 DX2 IBM computer. Data were collected using linear amplifiers for forward and side scatter and logarithmic amplifiers for FL1, FL2 and FL4. Samples were first run using isotype control or single fluorochrome-stained preparations for color compensation. For each sample, multiparametric data were acquired for 100,000 events.

### ELISA assays

CCL25 and CCL28 concentration was evaluated in breast milk, plasma, and saliva using commercial ELISA kits (R&D Systems, Minneapolis, MN) and following the procedures suggested by the manufacturer. Chemokine production was calculated from a standard curve of the corresponding recombinant human chemokine.

### Measurement of HIV-specific IgA

Breast milk concentration of IgA was measured after the thawed stored samples had been centrifuged at 250,00 RCF for 30 minutes at 4°C to remove the lipid layer [21]. All samples were processed in the same way without access to information about the other laboratory and clinical parameters. The supernatants were diluted 1∶5000 in sterile saline solution or PBS just before use. HIV-specific IgA in breast milk and saliva were measured using a modified Calypte Biomedical HIV-1 enzyme immunoassay (EIA) (Calypte Biomedical Corporation, Berkeley, CA) based on a recombinant HIV-1 envelope protein as previously described [20].

### Experiments in mice

1) **Cloning of CCL28** The CCL28 murine chemokine gene was digested with AgeI from the pORF-mCCL28 plasmid (InvivoGen, San Diego, CA, USA) and a blunt end was produced with T4 DNA polymerase. Following digestion with NheI, the resulting 410 bp fragment was inserted into pCpG expression vector (InvivoGen), which had been digested with ScaI/NheI.

2) **Recombinant VSV**. Recombinant viruses were recovered as described previously [27]. Briefly, BHK-21 cells were grown to 50% confluence and infected at a multiplicity of infection (MOI) of 10 with vTF7-3, a vaccinia virus expressing T7 RNA polymerase [28]. One hour after infection, cells were transfected with 10 µg of pVSVNX2 [29], kindly provided by John Rose (Yale University, New Haven, CT), 3 µg of pBS-N, 5 µg of pBS-P and 2 µg of pBS-L. After 48 h, cell supernatants were added to fresh BHK-21 cells and the recovery of recombinant infectious VSV was performed 48 h later and confirmed by development of cytopathic effect. The recombinant VSV were propagated in BHK-21 cells and stored at −80°C.

**3) Immunization procedures.** Inbred female BALB mice (Charles River Laboratories, Calco Italy), 6–8 weeks old, were utilized. Mouse colonies were maintained on a 12-h light-dark cycle in cages of 5 animals with water and food provided ad libitum. Different groups of mice (5 mice/group) were immunized as follows: group 1 = VSV alone (1×10^5^/mouse); group 2 = VSV (1×10^5^/mouse)+CCL28 (50 µg/mouse); group 3 = CCL28 alone (50 µg/mouse); group 4 = saline alone (negative control group). VSV was administrated intraperitoneally; CCL28 was administrated intramuscularly. Mice were immunized on day 0, boosted on day 14, and sacrificed on day 28.

**4) Immunohystochemistry analyses.** Tissues obtained from the colon of mice (2-cm specimens from the anus toward the left colon) were fixed in buffered-formalin for 24 hours at room temperature and then embedded in paraffin. Haematoxylin-eosin stained sections were used for histological evaluation. The evaluation of CD138+ plasmacells was performed on 3 µm paraffin embedded selides; the sections were dewaxed in xylene, rehydrated in an ascendent ethanol scale and pre-treated in a microwave oven (two cycles for 5 minutes each at 780W, in EDTA buffer, pH8). Endogen biotin and aspecific signals were blocked by using the appropriate reagents. For immunohistochemistry, a rat anti-mouse Syndecan-1 antibody (CD138, dilution 1∶250, R&Dsystems, USA) was used; slides were incubated for two hours at room temperature in a humid chamber; washed in PBS and then revealed by biotinylated anti rat IgG (dilution 1∶50, one hour incubation, Vector Laboratories, USA) followed by ZyMax Streptavidin HRP Conjugated (dilution 1∶500, 30 minutes incubation, Zymed, USA). The chromogen was 3,3′-diaminobenzidine free base (DAB). Negative controls were obtained by omission of the primary antibody. The quantitative evaluation of immunostaining, was performed by counting positive cells in 15 High-Power Fields (the mean of values was considered). The evaluation of IgA+ plasmacells was made on 3 µm paraffin embedded selides; the sections were dewaxed in xylene, rehydrated in an ascendent ethanol scale and pre-treated in a microwave oven (two cycles for 5 minutes each at 780W, in EDTA buffer, pH8). Endogen biotin and aspecific signals were blocked by using the appropriate reagents. For immunohistochemistry, a goat anti-mouse IgA (dilution 1∶400, AbD Serotec) was used; slides were incubated for two hours at room temperature in a humid chamber; washed in PBS and then revealed by biotinylated anti goat IgG (dilution 1∶50, one hour incubation, R&Dsystems, USA) followed by Streptavidin HRP Conjugated (ready to use, 30 minutes incubation, R&Dsystems, USA). The chromogen was 3,3′-diaminobenzidine free base (DAB).

### Statistical analyses

Parameters were compared across groups using nonparametric tests (Mann-Whitney) unless normally distributed, in which case two-tailed T-tests were used. Correlations were calculated using the Spearman rank order coefficient. Kaplan-Meier analysis and log-rank testse were used to compare mortality across groups. Statistical analyses were performed using the SPSS statistical package (SPSS Inc. Chicago, Illinois, USA).

## Results

### CCL28 and HIV-specific IgA in breast milk from HIV-infected women

**Figure 1:**
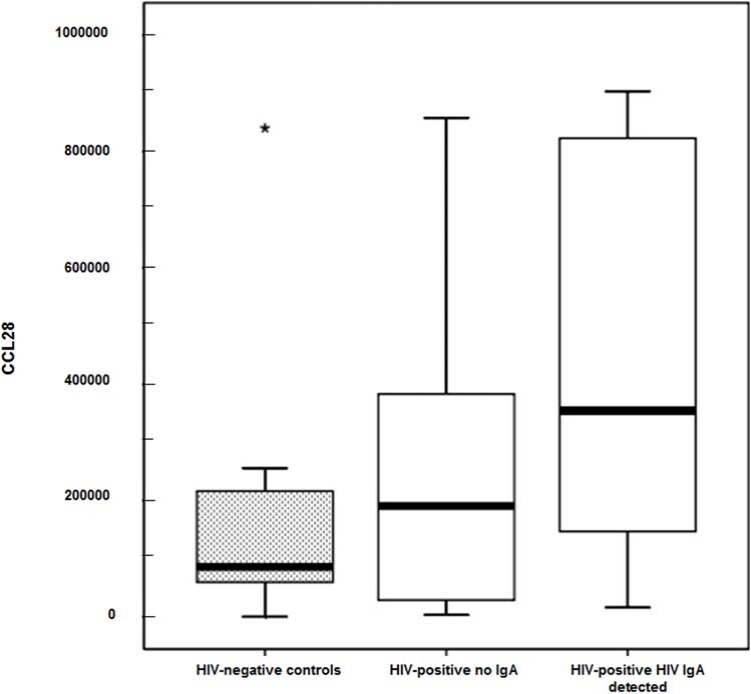
CCL28 concentration in breast milk of HIV-infected, HIV-exposed, and healthy women from Zambia. CCL28 concentration (pg/ml) in breast milk from Zambian women enrolled in the Zambia Exclusive Breastfeeding Study (ZEBS). Results from HIV-seronegative controls; HIV-infected women who did not have HIV-specific IgA in breast milk; and HIV-infected women with HIV-specific IgA in breast milk are shown. The boxes stretch from the 25^th^ to the 75^th^ percentile; the line across the boxes indicates the median value; the line stretching from the box reaches the largest and the smallest values detected in each group. Statistically significant differences are indicated.

CCL28 concentrations in breast milk were 2 log higher than that observed in saliva and almost 3 log higher than that observed in plasma Concentrations of CCL28 in breast milk were higher in milk from 65 HIV-infected women (median 288,480 pg/ml) than in milk from 9 uninfected women (median 86,170 pg/ml) but this did not reach significance (p = 0.11) presumably due to small numbers of uninfected women. Among HIV-infected women, CCL28 concentrations in breast milk were nevertheless significantly higher among 34 women who also had HIV-specific IgA detected in their milk (median 354,012 pg/ml) compared to 31 women who did not have HIV-specific IgA detected (median 190,640 pg/ml) (p = 0. 012) (Figure 1).

CCL28 concentrations in breast milk from HIV-infected women were not associated with maternal CD4 count, plasma viral load, or breast milk viral load. CCL28 concentrations in breast milk were not statistically different among 15 women who transmitted HIV to their infants (median 288,480 pg/ml) compared to 50 women who did not (median 291,227 pg/ml).

A significant association was nevertheless detected between CCL28 concentration in breast milk and survival of HIV-infected children. Among the 15 HIV-infected children, those 7 who died in the first 18 months of life had mothers with lower BM concentrations of CCL28 (median 146,995 pg/ml) than those 8 infants who survived past this age (median 349,472 pg/ml). Based on Kaplan-Meier survival curves, those HIV-infected children whose mothers had CCL28 concentrations less than median in the population (288,480 pg/ml) were more likely to die in the first 18 months of life than those whose mothers had CCL28 concentrations in breast milk greater or equal to the median (log rank p-value  = 0. 038).

### CCL28 in plasma and saliva of ESN, HC, and HIV individuals

**Figure 2:**
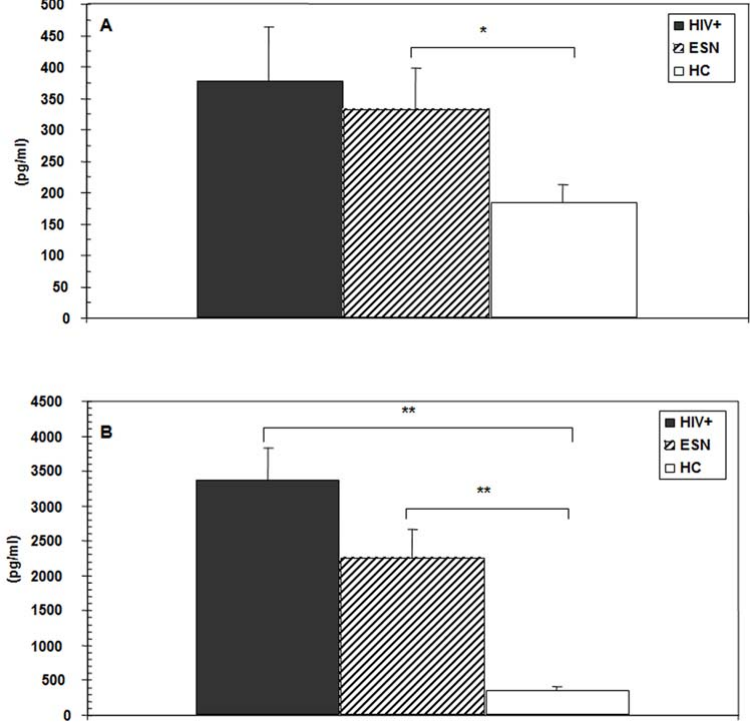
CCL28 concentration in plasma and saliva of HIV-infected, HIV-exposed, and healthy Italian individuals. CCL28 concentration (pg/ml) in plasma (A) and in saliva (B) of 39 HIV-exposed seronegative individuals (ESN), 40 HIV-infected individuals (HC), and 30 HIV-uninfected healthy controls (HC). Median values, standard deviations, and statistically significant differences are indicated.

CCL28 concentration was measured in plasma of 39 ESN, 40 HIV-infected individuals, and 30 HIV-uninfected healthy controls. Results showed that this IgA-secreting cells chemotactic factor was increased in plasma of ESN and HIV patients compared to HC. Thus, median CCL28 plasma concentrations were HIV = 379; ESN = 332 pg/ml; HC = 185 pg/ml (ESN vs. HC p = 0.039). These results are shown in Figure 2A. In contrast, no differences in the concentration of CCL25 were seen among the three groups of individuals examined (data not shown).

The concentration of CCL28 was also significantly increased in saliva of ESN and HIV individuals compared to healthy controls. Median CCL28 salivary concentrations were: HIV = 3379 pg/ml; ESN = 2259 pg/ml; HC = 364 pg/ml (HIV vs. HC p = 0.001; ESN vs. HC p = 0.007)(Figure 2B). CCL25 was comparable in ESN, HIV, and HC (data not shown).

### CCR3- and CCR10- expressing CD19+ cells in ESN, HC, and HIV individuals

**Figure 3:**
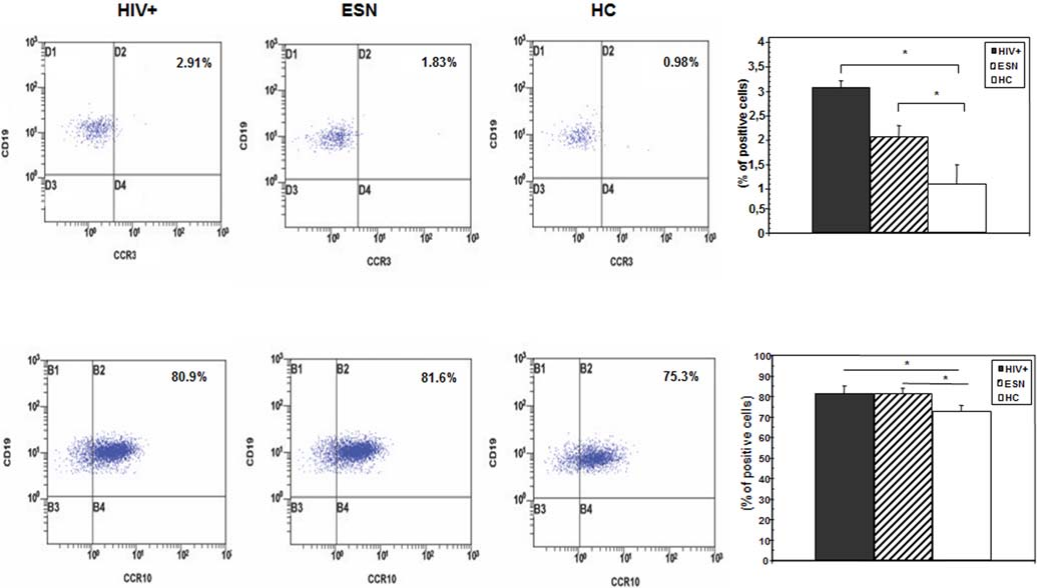
Expression of CCR3 and CCR10 on CD19+ B lymphocytes. Upper panels: CCR3-expressing, CD19+ B lymphocytes in peripheral blood. Lower panels: CCR10-expressing, CD19+ B lymphocytes in peripheral blood. Representative results as well as median values, standard deviations, and statistically significant differences are shown. Thirty-nine HIV-exposed seronegative individuals (ESN), 40 HIV-infected individuals (HC), and 30 HIV-uninfected healthy controls (HC) were analyzed.

Flow cytometry evaluation of CCR3 and CCR10 was performed in CD3+, CD14+ and CD19+ peripheral blood mononuclear cells. Results showed that the CCR3- and CCR10- expressing CD3+ and CD14+ cells were comparable in the three groups examined. In contrast, the percentage of CD19+/CCR3+ and of CD19+/CCR10+ lymphocytes were augmented in HIV patients (p = 0.027 and 0.024, respectively) and in ESN (p = 0.026 and 0.012, respectively) compared to HC (Figure 3). In contrast with these findings, CCR9-expressing lymphocytes were similar in all the individuals analyzed (data not shown).

### Surface density of CCR3 and CCR10 on CD19+ cells in ESN, HC, and HIV individuals

**Figure 4:**
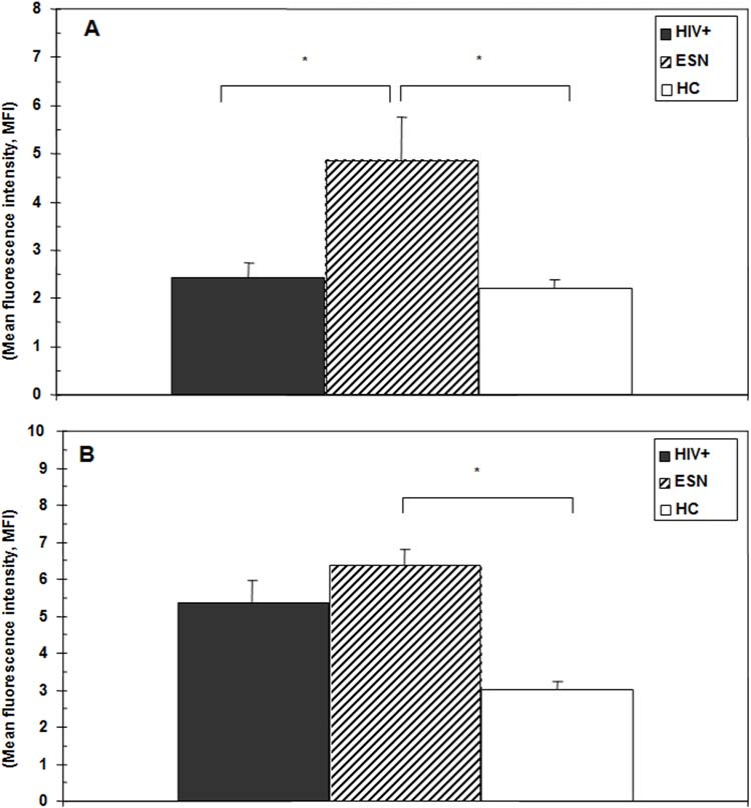
CCR3 and CCR10 mean fluorescence intensity on CD19+ B lymphocytes. Median fluorescence intensity (MFI). Panel A: CCR3+/CCD19+ B lymphocytes; panel B: CCR10+/CCD19+ B lymphocytes. Median values, standard deviations, and statistically significant differences are indicated. Thirty-nine HIV-exposed seronegative individuals (ESN), 40 HIV-infected individuals (HC), and 30 HIV-uninfected healthy controls (HC) were analyzed.

Evaluation of the mean fluorescence intensity (MFI), a relative measure of the surface density on a cellular level, revealed that CCR3 MFI on CD19+ cells was significantly augmented in ESN compared to both HIV patients (0.021) and HC (0.034) but was similar between HIV and HC subjects. CCR10 MFI on CD19+ lymphocytes was higher in ESN and HIV compared to HC (ESV vs. HC p = 0.019) (Figure 4). Also in this case, CCR9 MFI was comparable among all groups examined (data not shown).

### Salivary and BM concentration of CCL28 and HIV-specific IgA are correlated

CCL28 chemoattracts IgA-ASC in the MLP and in the mammary gland; mucosal HIV-specific IgA are observed in both HIV infection and exposure. We verified the presence of possible correlations between HIV-specific IgA and CCL28 both in saliva and BM in the individuals enrolled in the study. Results showed that a significant correlation indeed exists between these two parameters both in saliva (spearman rank order correlation coefficient r = 0.44 p = 0.016) and BM (spearman rank order correlation coefficient: r = 0.26 p = 0. 036).

### Administration of CCL28 augments rectal IgA in mice

The gastro-intestinal MLP is the first site of massive HIV replication during primary HIV infection; this phenomenon is associated with a dramatic early destruction of CD4+ memory T lymphocytes. Elicitation of adaptive mucosal immunity could therefore have an important beneficial effect in the prevention of the establishment of HIV infection. As the results herein showed that increased CCL28 are correlated with augmented concentrations of HIV-specific IgA, we verified whether immunization of mice with a VSV vector in the presence of CCL28 could have resulted in an increase of IgA-ASC in the GI MLP.

**Figure 5:**
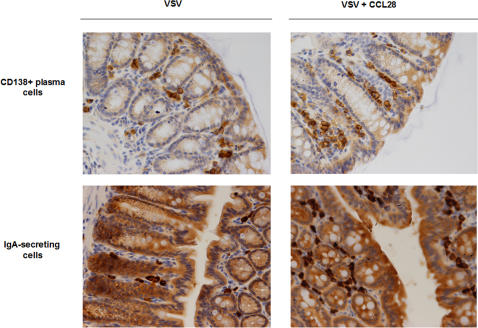
Effect CCL28 on colon plasmacells and IgA of VSV-immunized mice. Upper panels: CD138+ plasmacells; Lower panels: IgA+ plasmacells. Left column: Balb/c mice immunized with VSV alone; right column: Balb/c mice immunized with VSV in the presence of CCL28. 5 mice/group were vaccinated. Representative results obtained in the lamina propria of colon mucosa (2-cm specimens from the anus toward the left colon) are presented.

CD138+ plasmacells were clearly identified by a immunopositive staining at the cytoplasmic membrane. Positive cells were localized in the lamina propria of colonic mucosa, isolated or grouped in small clusters. Results, presented in Figure 5, demonstrated that the number of CD138+cells was higher in VSV-CCL28 mice than in saline, VSV-alone, or CCL28 animals. IgA+ plasmacells were identified by immunopositive staining in the cytoplam of cells with plasmacell-like morphology. In VSV+CCL28 mice, IgA+ cells were numerous and grouped in clusters within the lamina propria of colonic mucosa (mean number and S.D. of IgA+ plasmacells = 69±17; p vs. all other groups <0.001). In VSV-alone (34+7 IgA+ plasmacells), saline alone (22+15 IgA+ plasmacells), or CCL28 alone (31+13 IgA+ plasmacells) mice, only rare IgA+ cells were identified by immunohistochemistry; they were isolated in the lamina propria of the colon and not clustered. These results suggest a potential usefulness for CCL28 as a mucosal-directed adjuvant.

## Discussion

IgA are mostly mucosal antibodies that are responsible for the first line of defence of adaptive immunity against pathogens [30]–[34]. High concentrations of HIV-specific mucosal IgA have been described in both HIV-infected [16]–[19] and HIV-exposed but uninfected individuals [20]–[24]. HIV-specific IgA seen in these two groups were nevertheless shown to be qualitatively different: IgA of ESN recognize epitopes different from those seen in HIV-infected individuals [35], are capable of inhibiting virus transcytosis through epithelial layers in vitro [Bibr pone.0000969-Devito1] [36. 37], and have a potent neutralizing activity [38], [39]. These qualitative differences notwithstanding, IgA-secreting plasma cells (IgA-ASC) are chemo-attracted in the mucosal lamina propria by distinctive CC chemokines called CCL28, or MEC, and CCL25, or TECK [1]–[15]. In the attempt to verify whether a correlation does exist between augmented concentrations of mucosal IgA in HIV exposure/infection and increased quantities of CCL28, we measured CCL28, CCL25 and surface expression of CCL28 and CCL25-binding receptors in ESN, HIV-infected patients, and HC. Results shown here indicate that the CCL28-dependent, but not the CCL25-dependent chemotactic circuit is upregulated in HIV exposure and infection, therefore justifying the augmented mucosal concentrations of IgA seen in this condition.

CCL28 was measured in different mucosal secretions: breast milk and saliva. As CCL28 was repeatedly shown to play a role in the migration of IgA-ASC in mammary and salivary glands [1]–[15] it is not totally unexpected that the concentrations of this factor were higher in both sites in HIV infection and exposure, two conditions characterized by increased IgA concentrations. It could apparently be more surprising that CCL28 was augmented in serum, and that circulating CD19+ cells express more frequently, and at a higher density, CCR3 and CCR10. These results nevertheless become more clear considering that lymphocyte recirculation between tissues and blood compartments is a defining feature of the immune system. The up regulation of CCR3 and CCR10 observed on circulating CD19+ lymphocytes could thus be dependent on the recirculation of lymphocytes between the mucosal and haematic compartments Analogously, the augmented concentrations of CCL28 observed in the mucosal tissues were reflected in the higher quantities of plasmatic CCL28, that, although one fold lower than what is observed in saliva, still showed significant differences when ESN and HIV individuals were compared to healthy controls. It remains to be determined whether the expression of CCR3 and CCR10 in CD19+ lymphocytes isolated from mucosal secretions will be augmented as well in HIV infection and exposure.

IgA are the first and main defensive line used by adaptive immunity at the mucosal level. Not surprisingly, in HIV-infected patients, IgA, but not IgG prevent virus penetration into mucosal targets, thus prevent the establishment of infection [40]. In this light it seems important that CCL28 concentration in breast milk were positively correlated with better survival in Zambian breast-fed and vertically HIV-infected children. These children were enrolled in a protocol (Zambia Exclusive Breastfeeding Study; ZEBS) and randomized to either stop breastfeeding 4 months after birth or to continue breastfeeding. Recent analyses have shown that HIV-infected children who stop breastfeeding had a very high risk of dying shortly after interruption of breastfeeding (Sinkala, M., *et al.* No benefit of early cessation of breastfeeding at 4 months on HIV-free survival of infants born to HIV-infected mothers in Zambia: the Zambia Exclusive Breastfeeding Study. 14^th^ Conference on Retroviruses and Opportunistic Infections. February 25–28, 2007. Los Angeles, CA.) It could be argued that this mortality is secondary to withdrawal from the positive effect of BM-containing IgA and CCL28 at a time of life when the immune response is still immature. Finally, the correlation between CCL28 and HIV-specific IgA in BM described herein could also justify the observation that children who acquire HIV through breastfeeding have a better prognosis than children who acquire it at delivery or during pregnancy [41].

Recent studies have demonstrated that the gut associated lymphoid tissue (GALT) contains the vast majority of T cells in the body, and that GALT is the preferential target for HIV replication during the acute phase of HIV disease [42]–[44]. This phenomenon results in a massive and precocious destruction of CD4+ T cell that strongly impacts on the pathogenesis of HIV infection [42]–[44]. These observations have further strengthened the importance and urgency of developing mucosal vaccine for the prevention of HIV infection [45], [46]. Based on the result shown here, we decided to verify whether immunization in the presence of CCL28 would have had a significant effect on mucosal IgA at the gastro-intestinal (GI) level. This hypothesis was indeed fulfilled by the observation that IgA-ASC were significantly increased in the GI lamina propria of mice that were immunized with VSV in the presence of CCL28. Although preliminary, these results suggest that the use of CCL28 as an adjuvant could result in a beneficial modulation of mucosal immunity. Experiments are in progress in which the biologic effect of CCL28 is further analyzed in animal models.

Tissue-specific migration of lymphocytes is tightly regulated by a complex network of chemokines. CCL28 has been shown to direct homing of ASC to the gastro-intestinal and the upper aero-digestive as well as to the mammary glands [1]–[15]. CCL28 binds to CCR3 and CCR10; CCR10 in particular is considered to be a unifying chemokine receptor that plays a pivotal role in homing of plasmablasts to extra-intestinal effector sites [1]–[15]. The ability of the CCL28-CCR3/CCR10 circuit to chemoattract ASC into multiple mucosal sites has indeed been a founding element of the concept of a common mucosal immune system, and very recent results indicate that CCL28 has a broad and unifying role in the physiology of the mucosal IgA immune system. It might therefore not be surprising that the CCL28 but not the CCL25 circuit is upregulated in HIV infection and exposure.

The results herein confirm the hypothesis that CCL28 has a hierarchically dominant role in the modulation of adaptive immune responses at the mucosal level and suggest that CCL28 could be useful in the design of mucosal vaccine approaches finalized at the prevention of HIV infection.
